# Warning against Contagion from Consumption

**Published:** 1889-11

**Authors:** Emmons Clark

**Affiliations:** Secretary


### WARNING AGAINST CONTAGION FROM CONSUMPTION.

*From Peoria Medical Monthly.*

We are glad to note the following circular of warning, issued by the health department of the city of New York. It is based upon the conclusions arrived at by a committee of the best physicians and pathologists of that city, and may be safely said to be a move in the right direction.

We would be glad to see it circulated extensively by every board of health in the country, and physicians should be supplied with copies to be left in every household where consumption is found:

### RULES TO BE OBSERVED FOR THE PREVENTION OF THE SPREAD OF CONSUMPTION.

Pulmonary tuberculosis (consumption) is directly communicated from one person to another. The germ of the disease exists in the expectoration of persons afflicted with it. The following extract from the report of the pathologists of the health department explains the means by which the disease may be transmitted:

Tuberculosis is commonly produced in the lungs (which are the organs most frequently affected) by breathing air, in which living germs are suspended as dust. The material which is coughed up, sometimes in large quantities, by persons suffering from consumption, contains these germs in enormous numbers. * * This material when expectorated, frequently lodges in places where it dries, as on the street, floors, carpets, handkerchiefs, etc. After drying in one way or another, it is very apt to become pulverized and float in the air as dust.

By observing the following rules the danger of catching the disease will be reduced to a minimum:

1. Do not permit persons suspected to have consumption to spit on the floor or on cloths, unless the latter be immediately burned. The spittle of persons suspected to have the consumption should be caught in earthen or glass dishes containing the following solution: Corrosive sublimate 1 part, water 1,000 parts.

2. Do not sleep in a room occupied by a person suspected of having consumption. The living rooms of a consumptive patient should have as little furniture as practicable. Hangings should be especially avoided. The use of carpets, rugs, etc., ought always to be avoided.

3. Do not fail to wash thoroughly the eating utensils of a person suspected of having consumption as soon after eating as possible, using boiling water for the purpose.

4. Do not mingle the unwashed clothing of consumptive patients with similar clothing of other persons.

5. Do not fail to catch the bowel discharges of consumptive patients with diarrhœa in a vessel containing corrosive sublimate 1 part, water 1,000 parts.

6. Do not fail to consult the family physician regarding the social relations of persons suffering from suspected consumption.

7. Do not permit mothers suspected of having consumption to nurse their offspring.

8. Household pets (animals or birds) are quite susceptible to tuberculosis; therefore do not expose them to persons afflicted with consumption; also do not keep, but destroy at once, all household pets suspected of having consumption, otherwise they may give it to human beings.

9. Do not fail to thoroughly cleanse the floors, walls and ceilings of the living and sleeping rooms of persons suffering from consumption at least once in two weeks.

By order of the Board.

EMMONS CLARK,
Secretary.

